# Behavioral evolution accompanying host shifts in cactophilic *Drosophila* larvae

**DOI:** 10.1002/ece3.4209

**Published:** 2018-06-11

**Authors:** Joshua M. Coleman, Kyle M. Benowitz, Alexandra G. Jost, Luciano M. Matzkin

**Affiliations:** ^1^ Department of Entomology University of Arizona Tucson Arizona; ^2^ Department of Biological Sciences University of Alabama in Huntsville Huntsville Alabama; ^3^ BIO5 Institute University of Arizona Tucson Arizona; ^4^ Department of Ecology and Evolutionary Biology University of Arizona Tucson Arizona

**Keywords:** activity, cactophilic, *Drosophila mojavensis*, larval locomotion, local adaptation, plant structure, pupation

## Abstract

For plant utilizing insects, the shift to a novel host is generally accompanied by a complex set of phenotypic adaptations. Many such adaptations arise in response to differences in plant chemistry, competitive environment, or abiotic conditions. One less well‐understood factor in the evolution of phytophagous insects is the selective environment provided by plant shape and volume. Does the physical structure of a new plant host favor certain phenotypes? Here, we use cactophilic *Drosophila*, which have colonized the necrotic tissues of cacti with dramatically different shapes and volumes, to examine this question. Specifically, we analyzed two behavioral traits in larvae, pupation height, and activity that we predicted might be related to the ability to utilize variably shaped hosts. We found that populations of *D. mojavensis* living on lengthy columnar or barrel cactus hosts have greater activity and pupate higher in a laboratory environment than populations living on small and flat prickly pear cactus cladodes. Crosses between the most phenotypically extreme populations suggest that the genetic architectures of these behaviors are distinct. A comparison of activity in additional cactophilic species that are specialized on small and large cactus hosts shows a consistent trend. Thus, we suggest that greater motility and an associated tendency to pupate higher in the laboratory are potential larval adaptations for life on a large plant where space is more abundant and resources may be more sparsely distributed.

## 1 INTRODUCTION

Insects utilizing plant tissues, both living and necrotic, have undergone one of the most successful and expansive radiations of any group of organisms (Bernays & Chapman, 1994; Thompson, 1968; Throckmorton, 1975; Wiens, Lapoint, & Whiteman, 2015). Many authors have attributed the success of these groups to the ability of insects to rapidly colonize novel hosts (Funk & Nosil, 2008; Linnen & Farrell, 2010; Oliveira et al., 2012; Winkler & Mitter, 2008). Therefore, understanding the depth and complexity of phenotypic adaptations made by insects utilizing new hosts is essential to understanding their diversity. Hypotheses for the basis of such adaptations have largely focused around plant chemistry, and the ability of insects to survive in and utilize novel chemical environments (Becerra, 1997; Ehrlich & Raven, 1964; Futuyma & Agrawal, 2009).

One ecological variable that has received considerably less attention is the physical structure of the host plant, such as its shape, volume, and more importantly the usable resource distribution within the host. While adult insects are known to use aspects of plant or fruit shape as cues for oviposition on recently adapted hosts (Alonso‐Pimentel, Korer, Nufio, & Papaj, 1998; Kanno & Harris, 2000; Prokopy, 1968), it is unclear how frequently insects have adapted specifically to maximize fitness on plants of different physical structure. Among the traits that potentially are influenced by the shape, volume, and size of the host plant are the foraging behavior of larvae. Adult foraging behavior has long been thought to be controlled by habitat structure (Moermond, 1979; Robinson & Holmes, 1982; Uetz, 1991). For insects, especially holometabolous insects, host structure is unlikely to define the foraging environment for adults, which is more likely related to the distribution of plants throughout the broader landscape. However, for larvae that can only crawl, the shape and volume of the host plant should present strict boundaries to the available foraging habitat. Therefore, we hypothesize that variation in the physical structure of the host plant, *that is,* its shape and volume, should influence larval insect behaviors related to foraging or motility.

Cactophilic *Drosophila* have long served as a model system for examining adaptations associated with evolutionary shifts in host plant usage (Matzkin, 2014). Populations of one well‐studied species, *D. mojavensis*, utilize different cactus species as hosts in four geographically distinct populations: prickly pear (*Opuntia littoralis*) on Santa Catalina Island, agria (*Stenocereus gummosus*) (and to a lesser extent cochal, *Myrtillocactus cochal*) in Baja California, red barrel cactus (*Ferocactus cylindraceus*) in the Mojave Desert, and organ pipe (*S. thurberi*) as well as occasionally cina (*S. alamosensis*) in the Sonoran Desert. Larval and adult *D. mojavensis* feed on yeast (Fogleman, Starmer, & Heed, 1981, 1982) and bacteria (Fogleman & Foster, 1989) present in the necrotic tissues of these cacti. Cactus host adaptation across these populations (reviewed in Matzkin, 2014) have shaped variation at detoxification pathways (Matzkin, 2005, 2008), life history characteristics (Etges, 1993; Etges & Heed, 1987; Rajpurohit, Oliveira, Etges, & Gibbs, 2013), behavior (Etges, Over, De Oliveira, & Ritchie, 2006; Newby & Etges, 1998), and morphology (Pfeiler, Castrezana, Reed, & Markow, 2009) as well as more broadly at the genomic and transcriptomic (Matzkin & Markow, 2013; Matzkin, Watts, Bitler, Machado, & Markow, 2006) level. Furthermore, additional closely related cactophilic species within the *D. repleta* species group have colonized variable cactus environments (Oliveira et al., 2012), providing the potential for deeper evolutionary comparisons.

In addition to displaying extensive chemical and microbial variation (Fogleman & Abril, 1990; Kircher, 1982; Starmer, 1982; Starmer & Phaff, 1983), the cactus species inhabited by *D. mojavensis* also have striking physical differences, displaying marked variation in shape, volume, and size. Among the major columnar species used, *S. thurberi* (utilized in Sonora) is typically the taller species (4–7 m) and has numerous thick arms (15–20 cm; Gibson & Horak, 1978; Turner, Bowers, & Burgess, 2005). The species mainly utilized in Baja California, *S. gummosus*, is shorter (3–5 m) with many thinner arms (5–10 cm; Gibson & Horak, 1978; Cody, 1984; Turner et al., 2005). Red barrel cactus, (*F. cylindraceus*), is shaped much differently, with a single short (<1.5 m) but wide (30–40 cm) rounded stem (McIntosh, 2002). The prickly pear (*O. littoralis*) plant is also short (<1 m), but consists of numerous small, elliptical flat cladodes (20–30 cm long, 10–15 cm wide and 2–4 cm thick) (Benson & Walkington, 1965). Other related cactophilic *Drosophila* species beyond *D. mojavensis* have colonized additional cactus species with variable physical characteristics. *Drosophila arizonae*, the sister species of *D. mojavensis*, is a cactus generalist throughout its range (Sonoran Desert to southern Mexico as well as the Baja California peninsula and more recently in southern California), occupying various species of both prickly pear and columnar cactus (Fellows & Heed, 1972; Heed, 1978, 1982). *Drosophila navojoa* previously collected from southern Sonora and Jalisco specializes on prickly pear (*O. wilcoxii*) necrotic cladodes and fruits (Heed, 1982). *Drosophila nigrospiracula* mainly utilizes the giant saguaro (*Carnegiea gigantea*; Fellows & Heed, 1972; Heed, 1982), and cardón (*Pachycereus pringlei*), both tall cacti (12 m and 20 m, respectively) with thick stems (20–40 cm and 40–150 cm, respectively) and multiple arms present in the Sonoran desert (Gibson & Horak, 1978; Turner et al., 2005).

If the shape and volume of the necrotic cactus resources define the boundaries of the foraging environment for larvae, we predicted that movement‐related behaviors should differ between flies inhabiting cacti of different shapes, and specifically that the behavior of larvae native to larger and longer columnar cacti should reflect an ability to forage across greater distances, potentially allowing access to additional or preferable sources of nutrition. Conversely, individual larvae utilizing prickly pear cladodes will be restricted by the size of cladode itself, limiting the need to travel long distances to forage.

To test these predictions, we quantified pupation height and third‐instar speed across the four *D. mojavensis* populations under common garden conditions. We found that flies from the Catalina Island population (prickly pear) were both slower and pupated closer to the food resource than flies from the other populations, especially the Sonoran population (columnar). Furthermore, the specialist species *D. navojoa* and *D. nigrospiracula*, which inhabit primarily small (prickly pear) and large cactus (saguaro and cardón), respectively, display consistent results for larval speed. The generalist *D. arizonae* displays intermediate phenotypes for both pupation height and speed. Lastly, F_1_ crosses between the Catalina Island and Sonoran populations suggest that speed and pupation height are genetically independent phenotypes in *D. mojavensis*. We argue that both phenotypes are likely related to the shape, volume, and size of the host cacti.

## 2 MATERIALS AND METHODS

### 2.1 Experimental insects

We utilized isofemale lines of *D. mojavensis* originally collected from Santa Catalina Island, the Sonoran Desert (Organ Pipe National Monument, AZ), Baja California (La Paz, Mexico), and the Mojave Desert (Whitman Canyon, AZ and Anza Borrego, CA), on the cacti described above. We also used isofemale lines of the generalist species *D. arizonae* originally collected from Baja California (San Pedro, Mexico), Southern California (Riverside, CA), southern Mexico (Hidalgo and Chiapas), and the Sonoran Desert (Tucson, AZ, Hermosillo, Mexico, and San Carlos, Mexico). Lastly, we used a multifemale stock of *D. nigrospiracula* collected from Saguaro (*Carnegiea gigantea*) in Tucson, AZ, and a *D. navojoa* stock originally collected on prickly pear (*Opuntia spp*.) from Jalisco, Mexico, from the Drosophila Species Stock Center (15081‐1374.11). Additional information on fly stocks and collections can be found under Dryad accessionhttps://doi.org/10.5061/dryad.j34g342. With the exception of *D. nigrospiracula*, we maintained all lines on banana‐molasses media (Appendix 1), at 25°C, 50% humidity, and a 14:10 light:dark cycle. *Drosophila nigrospiracula* was maintained on potato flake media with a necrotic saguaro homogenate mixture (Castrezana, 1997).

We quantified both pupation height and larval activity in isofemale lines of each of the four *D. mojavensis* populations. We measured pupation height from the Baja California population of *D. arizonae*. We also measured larval speed in lines from four *D. arizonae* populations as well as *D. navojoa* and *D. nigrospiracula*. We further examined both phenotypes in F_1_ individuals from a cross between Catalina Island (genome stock, 15081‐1352.22) and Sonoran Desert (MJ122) lines. To generate F_1_ larvae, virgin adults were collected from the Sonora and Catalina Island lines within 24 hr of eclosion. Virgin females from Sonora were then crossed with virgin males from the Catalina Island population in a cage with banana agar medium. The reciprocal cross was done in the same manner. F_1_ larvae were reared in the conditions described above until their use in either pupation or activity trials.

### 2.2 Larval activity assays

To assay larval activity, we placed 7–10‐day old virgin flies in mixed sex vials with the appropriate media under a 14:10‐hr light:dark cycle at 50% humidity and 25°C for 24 hr before removing all adults and allowing eggs to hatch and develop undisturbed. Upon reaching the third instar, we removed larvae from vials in randomly selected groups of five and placed them on a 10 cm petri dish partially filled with 1% agar. Larvae in the third‐instar stage were determined by body size and used irrespective of specific age, because species as well as the four *D. mojavensis* populations vary substantially in developmental time (Etges, 1990; J.M.C. pers. obs.). We then recorded each group of larvae for 5 min using a Point Grey video camera (FLIR Systems, Wilsonville, OR, USA), and retained images taken every 5 s. To ensure that we disregarded an initial period of low activity after transfer, we utilized only the 50 s before each larva reached the wall of the experimental chamber (whereupon estimating the position of the larva became imprecise) for analysis. We analyzed the mean speed of each larva during this 50 sec period using the TrackMate plugin of the ImageJ software package (https://imagej.nih.gov/ij/). All activity trials were performed in full light conditions, in the afternoon between 12:00 and 3:00 p.m.

### 2.3 Pupation height assays

To measure pupation height, mated flies from stocks (see above) were maintained at low density in glass 8‐dram vials with banana‐molasses media and allowed to oviposit for 24 hr before being removed. Eggs were allowed another 24 hr to hatch into first instar larvae before being collected. Using a needle, 40 newly hatched larvae were placed in fresh 95 mm tall 8‐dram glass vials containing approximately 10 ml of banana‐molasses medium. Vials were capped using a packed cotton plug (Genesee Scientific), and then incubated at 25°C in 50% humidity on a 14:10 hr light:dark cycle. Larvae were then allowed to develop without disturbance. Once the larvae pupated, the distance between the surface of the food and the highest tip of the pupae was measured in millimeters using a digital calliper.

### 2.4 Statistical analyses

We analyzed pupation height data using GLMs modeled with quasipoisson error structures to account for non‐normality of the data, which contained a high number of zero values. We analyzed larval speed data using GLMs modeled with gaussian error structures. We used Tukey's HSD from the *multcomp* package (Hothorn, Bretz, & Westfall, 2008) in R to perform all pairwise post hoc comparisons for each GLM. We calculated Pearson's coefficient to estimate correlations between mean pupation height and mean speed across isofemale lines within the *D. mojavensis* Catalina Island and Sonora populations. To assess the effects of genotype on each phenotype, we analyzed isofemale line as a nested effect within population using the *lme4* package (Bates, Mächler, Bolker, & Walker, 2015) in R. We then used the ANOVA function to compare the performance of the mixed models to identical models with the nested term removed. All statistical analyses were performed in R 3.4.0 (https://www.R-project.org).

## 3 RESULTS

### 3.1 Phenotypic differences across populations and species

**Figure 1:**
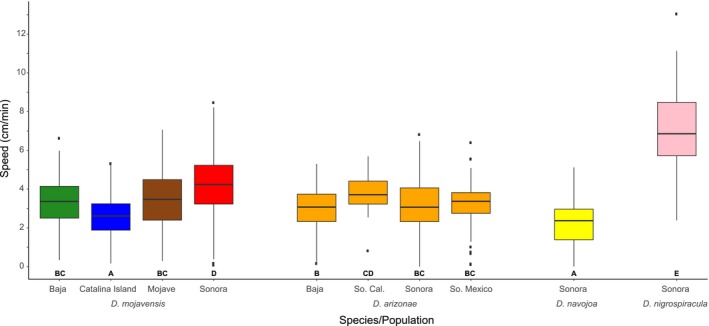
Third‐instar speed in four *D. mojavensis* populations, four *D. arizonae* populations, and single *D. navojoa* and *D. nigrospiracula* populations. Letters below each box indicate significant differences between species and populations. *D. mojavensis–*Baja *n* = 180, Catalina Island *n* = 279, Mojave *n* = 275, Sonora *n* = 280; *D. arizonae*––Baja *n* = 196, Southern California *n* = 35, Sonora *n* = 463, Southern Mexico *n* = 143; *D. nigrospiracula*––*n* = 60; *D. navojoa*––*n* = 108

Larval speed varied significantly both between *D. mojavensis* populations and across species (Figure 1). Catalina Island flies were significantly slower than flies from Baja, Mojave, or Sonora. Baja and Mojave populations were not different, though Sonora was different from both (Appendix 2). All populations of *D. arizonae* larvae displayed intermediate speeds. However, *D. navojoa* and *D. nigrospiracula* showed extreme phenotypes, the former being slower than all but Catalina Island *D. mojavensis* while the latter was significantly faster than all other species and populations. Isofemale lines of *D. mojavensis* also exhibited significant genetic variation (*χ* ^2^ = 256.74, *p* < 0.0001; Appendix 3).

**Figure 2:**
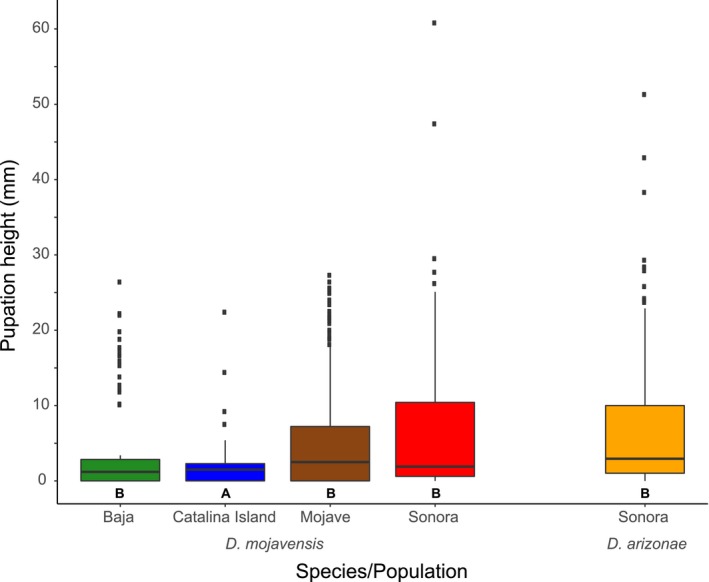
Pupation height in four *D. mojavensis* populations and one *D. arizonae* population. Letters below each box indicate significant differences between species and populations. *D. mojavensis–*Baja *n* = 84; Catalina Island *n* = 399, Mojave *n* = 288, Sonora *n* = 340; *D. arizonae–*Sonora *n* = 398

Pupation height also displayed significant variability across *D. mojavensis* populations (Figure 2). Flies from Catalina Island pupated at lower height than flies from any of the other three populations or the sister species *D. arizonae*, none of which displayed significant differences in pupation height between them (Appendix 4). Isofemale lines of *D. mojavensis* exhibited significant genetic variability (*χ* ^2^ = 145.09, *p* < 0.0001; Appendix 3).

Genetic correlations between *D. mojavensis* pupation height and third‐instar speed were not significantly different from zero within either the Catalina Island population (*r*_10_ = −0.241; *p* = 0.475) or within the Sonora population (*r*_9_ = 0.203; *p* = 0.578).

### 3.2 Phenotypes of F_1_ crosses

**Figure 3:**
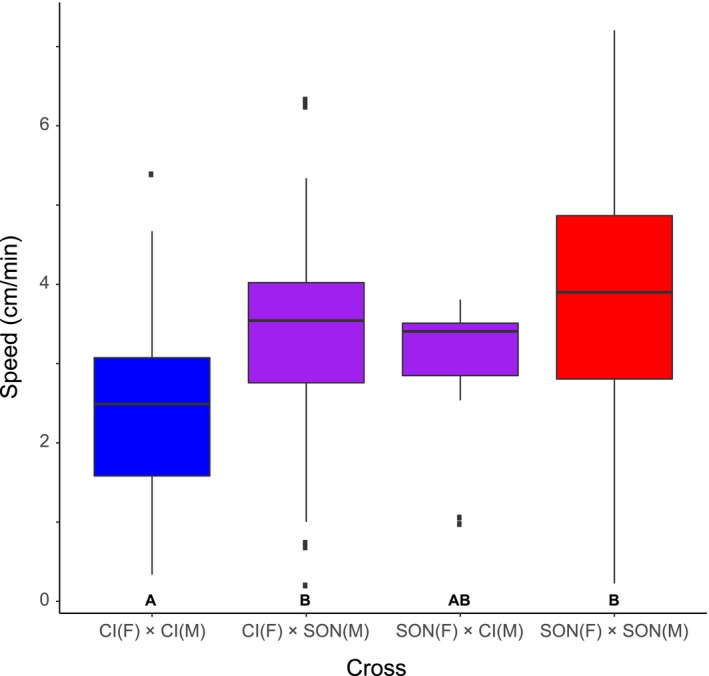
Third‐instar speed in parental lines and F_1_ crosses between Catalina Island and Sonora populations of *D. mojavensis*. Letters below each box indicate significant differences between groups. Catalina Island *n* = 60; Catalina Island (F) × Sonora (M) *n* = 113; Sonora (F) × Catalina Island (M) *n* = 15; Sonora *n* = 113

Third‐instar speed of the Sonoran population was not significantly different from either F_1_ cross, but was, as expected, greater than the Catalina Island (Figure 3). Speed of the Catalina Island population was slower than the Catalina female by Sonora male cross but not the reciprocal. The F_1_ crosses were also not different from each other (Appendix 5).

**Figure 4:**
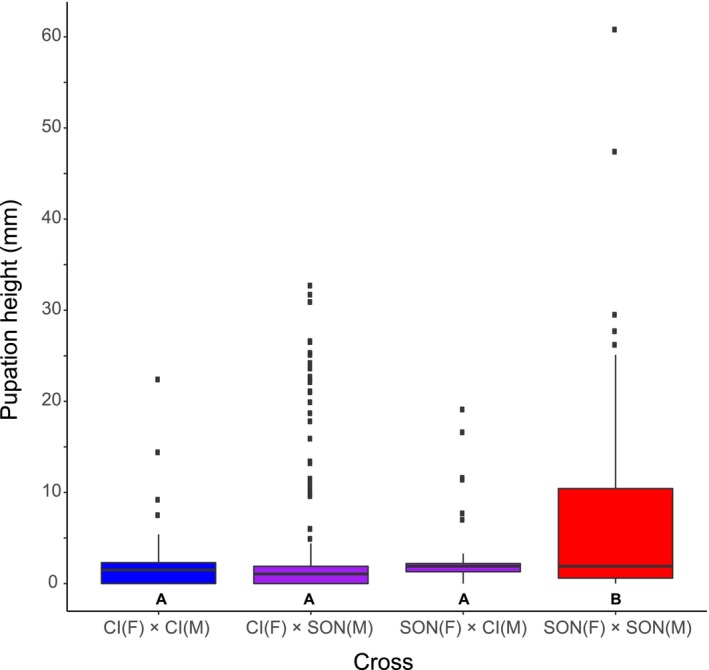
Pupation height in parental lines and F_1_ crosses between Catalina Island and Sonora populations of *D. mojavensis*. Letters below each box indicate significant differences between groups. Catalina Island *n* = 399; Catalina Island (F) × Sonora (M) *n* = 644; Sonora (F)  × Catalina Island (M) *n* = 366; Sonora *n* = 340

The Sonoran population had significantly higher pupation heights than Catalina Island flies or either F_1_ cross (Figure 4). Both F_1_ crosses and Catalina Island populations displayed no difference among them in pupation (Appendix 6).

## 4 DISCUSSION

The hosts used by phytophagous and saprophytic insects display dramatic phenotypic variation along several axes. Many of these traits, including chemical traits, have been found to incur selective pressures on their insect cohabitants, leading to local adaptation (Bernays & Chapman, 1994; Thompson, 1968; Throckmorton, 1975; Wiens et al., 2015). However, physical characteristics such as shape, volume, and size of host plants or usable resource within a host plant (e.g., necrotic section) have seldom been investigated for their role in creating novel selective environments for insects. For insect larvae, the shape and volume of the host on which eggs are oviposited should constrain their movement, defining the boundaries of their foraging environment. Thus, we considered larval behavioral phenotypes as strong candidates for local adaptation to plants with variable physical characteristics. To examine this prediction, we measured pupation height and third‐instar activity in four populations of *D. mojavensis*, which has colonized multiple cactus host species throughout southwestern North America.

Larval speed was greater in *D. mojavensis* populations living on taller, larger cacti, and lowest in the Catalina Island population inhabiting necrotic prickly pear cladodes. Interspecific data also support this relationship, as *D. nigrospiracula* specializing on the saguaro and cardón cactus have very fast larvae, while *D. navojoa* inhabiting prickly pear are especially slow. Larval speed has generally been interpreted as a foraging related trait in *Drosophila*, exemplified by the rover/sitter polymorphism of *D. melanogaster* (Sokolowski, 1980). Furthermore, Sokolowski (1980) has suggested that increased speed may be an adaptation to widespread or discontinuous food sources. This type of distribution is likely to be a characteristic of the larger columnar arms of organ pipe, agria, and saguaro, where necroses are known to occur in patches at the ends of arms (Nobel, 1980). Organ pipe cactus has been previously observed to be associated with the lowest egg‐to‐adult viability when flies from Sonora and other populations are reared on it (Date, Crowley‐Gall, Diefendorf, & Rollmann, 2017; Etges & Heed, 1987). This could partly explain the pattern of increased motility of Sonoran larvae, but not for all populations (e.g., agria rots are of better quality, but Baja California larvae are among the fastest). On the small cladodes of prickly pear, slow speed may be advantageous because of energetic costs (Roff, 2002) when the potential advantages of high motility and long foraging distances have been removed. Furthermore, increased larval activity on a smaller resource might prove detrimental given that a potential severe fitness cost would be imposed to those larvae that wander out of the necrotic host resource. However, it is unlikely that cactus shape is the lone selective pressure shaping larval speed differences. Other environmental factors potentially influencing speed between species and populations might include temperature, toxicity, nutritional composition, competition between con‐ or heterospecific larvae, predation or parasitism. High toxicity is expected to select for slower speeds (Borash, Teotonio, Rose, & Mueller, 2000; Mueller et al., 2005), and because there are differences in chemical composition of columnars and prickly pear cacti (Kircher, 1982; Stintzing & Carle, 2005), this represents another environmental factor which could affect speed differences.

Pupation height also differed between *D. mojavensis* populations; larvae from larger columnar or barrel cacti pupated higher than larvae from shorter cacti, specifically, those living on prickly pear. As with speed, this behavioral difference may be related to the ability to efficiently utilize space. Studies of pupation in a variety of *Drosophila* species have attributed pupation location behavior to a variety of environmental factors, including biotic conditions such as conspecific density (Beltrami, Medina‐Muñoz, Arce, & Godoy‐Herrera, 2010; Sokal, Ehrlich, Hunter, & Schlager, 1960) and abiotic factors such as temperature (Dillon, Wang, Garrity, & Huey, 2009), light (Manning & Markow, 1981), moisture (Sameoto & Miller, 1968), and chemical composition (Beltrami et al., 2010). Therefore, pupation behavior in a given necrotic cactus host may reflect the ability of a larvae to take advantage of increased opportunities to find optimal pupation sites and mitigate the many biotic and abiotic risks listed above. We suggest that it is the ability to pupate higher, rather than actual pupation height, that may be advantageous on larger cacti. This could help explain the broad distribution of pupation phenotypes in the columnar and barrel *D. mojavensis* populations, all of which contain many individuals which pupate at low heights.

Variation in speed or pupation height might also be related to experimental conditions. It has been predicted that inbreeding should result in slower larval speeds in *Drosophila* (Bauer & Sokolowski, 1984). Consistent with this explanation in our dataset is the fact that *D. nigrospiracula*, the most active species, is also by far the most recently introduced to the laboratory, and therefore the most outbred. However, among the *D. mojavensis* populations, the slow Catalina Island population isofemale lines were actually the most recently established in the laboratory, though all have been maintained for well over the 20 generations of inbreeding required to purge nearly all genetic variation in *Drosophila* (Falconer & Mackay, 1996; Huang et al., 2014). Furthermore, the Catalina Island population has a significantly lower level of segregating variation and effective population size compared with the other three *D. mojavensis* populations (Machado, Matzkin, Reed, & Markow, 2007; Matzkin, 2004; Matzkin & Eanes, 2003; Reed, Nyboer, & Markow, 2007) and hence, the possible effect of inbreeding would be the least in this population. Also, the overestimation of phenotypic differences between isofemale lines is only a problem when lines are maintained with small numbers of individuals or for a handful (<10) generations (Hoffmann & Parsons, 1988), neither of which apply to our data. Thus, though inbreeding may be influencing pupation height or larval activity broadly, it is not likely leading to the specific pattern of differences reported here.

An additional possibility regarding pupation is that larval speed and pupation height are not truly distinct behavioral phenotypes. Might larvae pupate higher simply because their activity is greater, thus simply increasing the chances of being higher at the time of pupation? This would also be consistent with the broad distribution of pupation heights in the columnar/barrel *D. mojavensis* populations. However, we present several lines of evidence suggesting that this is not the case. First, we crossed the Catalina Island and Sonoran populations, which had the most extreme phenotypes for both traits. While crosses indicated a quantitative basis for both characters, both F_1_ hybrids displayed pupation height phenotypes insignificantly different from the Catalina Island parental population. This suggests a dominance effect to the alleles governing pupation height that we did not observe for third‐instar speed. Second, though sample sizes were small, we observed no evidence for a positive correlation of pupation height and speed within either the Catalina Island or Sonora populations. If pupation height was simply a consequence of increased speed, then lines within a population with higher speed should also exhibit higher pupation heights. Lastly, despite its remarkably high speed, previous work on *D. nigrospiracula* suggests that it does not pupate especially high compared to other cactophilic species (Fogleman & Markow, 1982). This also matches findings that pupation height and larval speed have distinct genetic bases in *D. melanogaster* (Bauer & Sokolowski, 1985; Sokolowski, 1985). Therefore, we suggest that speed and pupation height are truly separate traits potentially responding to environmental conditions on different cactus hosts.

We argue that host physical structure represents a strong candidate for a selective environment acting on both larval activity and pupation behavior. Given the importance of larval feeding on adult fitness, we expect host shape, volume, and size to impart similar selection pressures on other phytophagous and saprophytic insects and predict that activity and other traits related to resource utilization should consistently differ in larvae when insects have undergone host shifts to resources with novel physical characters.

## CONFLICT OF INTERESTS

The authors have no conflict of interests to declare.

## AUTHOR CONTRIBUTIONS

J.M.C., A.G.J., and L.M.M. conceived and designed research. J.M.C. and A.G.J. performed research. K.M.B. analyzed the data. K.M.B. and L.M.M. wrote the manuscript with input from all authors. All authors approved the final version of the manuscript.

## APPENDIX 1

### Recipe for banana‐molasses medium

De‐ionized water: 1 L

Drosophila agar type II: 14.17 g

Peeled banana: 137.4 g

Light corn syrup: 47.5 g

Molasses: 30 g

Baker's Yeast: 27.5 g

De‐ionized water for blending: 83.3 ml

Methylparaben (Tegosept): 2.23 g

190 proof ethanol: 22.3 ml

Add agar to deionized water and bring to a boil. Mixture is boiled for 15 min. Bananas and deionized water are blended till smooth. Light corn syrup, molasses, and baker's yeast are blended in. This is added to the boiling agar and is boiled for an additional 15 min. Media is cooled on ice until it reaches 54°C. Methylparaben is dissolved in 190 proof ethanol and added to the cooled media. Fly food is then portioned out into vials using a peristaltic pump. Media is stored at 4°C, and a few fresh yeast granules are added to each vial before use.

## APPENDIX 2

### Statistical tests of individual comparisons of third instar speed between populations, using Tukey's HSD

#### 1

**Table:**

Species	Population	*D. mojavensis*				*D. arizonae*				*D. navojoa*	*D. nigrospiracula*
		Baja	Catalina Island	Mojave	Sonora	Baja	Southern California	Sonora	Southern Mexico	Navojoa	Tucson
*D. mojavensis*	Baja										
	Catalina Island	***z*** ** = 5.712, ** ***p*** ** < 0.001**									
	Mojave	*z* = 1.547, *p* = 0.860	***z*** ** = 8.109, ** ***p*** ** < 0.001**								
	Sonora	***z*** ** = 7.795, ** ***p*** ** < 0.001**	***z*** ** = 15.250, ** ***p*** ** < 0.001**	***z*** ** = 6.954, ** ***p*** ** < 0.001**							
*D. arizonae*	Baja	*z* = 2.052, *p* = 0.536	***z*** ** = 3.607, ** ***p*** ** < 0.01**	***z*** ** = 3.842, ** ***p*** ** < 0.01**	***z*** ** = 10.301, ** ***p*** ** < 0.001**						
	Southern California	*z* = 2.307, *p* = 0.358	***z*** ** = 5.440, ** ***p*** ** < 0.001**	*z* = 1.537, *p* = 0.864	*z* = 1.793, *p* = 0.7183	***z*** ** = 3.483, ** ***p*** ** = 0.016**					
	Sonora	z = 0.923, *p* = 0.995	***z*** ** = 6.147, ** ***p*** ** < 0.001**	*z* = 2.998, *p* = 0.073	***z*** ** = 10.935, ** ***p*** ** < 0.001**	*z* = 1.538, *p* = 0.864	*z* = 2.898, *p* = 0.095				
	Southern Mexico	*z* = 0.129, *p* = 0.999	***z*** ** = 5.470, ** ***p*** ** < 0.001**	*z* = 1.306, *p* = 0.946	***z*** ** = 7.131, ** ***p*** ** < 0.001**	*z* = 2.063, *p* = 0.528	*z* = 2.186, *p* = 0.440	z = 1.001, *p* = 0.991			
*D. navojoa*	Navojoa	***z*** ** = 6.597, ** ***p*** ** < 0.001**	*z* = 2.242, *p* = 0.402	***z*** ** = 8.354, ** ***p*** ** < 0.001**	***z*** ** = 13.696, ** ***p*** ** < 0.001**	***z*** ** = 4.941, ** ***p*** ** < 0.001**	***z*** ** = 6.330, ** ***p*** ** < 0.001**	***z*** ** = 6.768, ** ***p*** ** < 0.001**	***z*** ** = 6.425, ** ***p*** ** < 0.001**		
*D. nigrospiracula*	Tucson	***z*** ** = 20.389, ** ***p*** ** < 0.001**	***z*** ** = 25.212, ** ***p*** ** < 0.001**	***z*** ** = 20.225, ** ***p*** ** < 0.001**	***z*** ** = 16.125, ** ***p*** ** < 0.001**	***z*** ** = 22.069, ** ***p*** ** < 0.001**	***z*** ** = 12.304, ** ***p*** ** < 0.001**	***z*** ** = 22.776, ** ***p*** ** < 0.001**	***z*** ** = 19.694, ** ***p*** ** < 0.001**	***z*** ** = 23.900, ** ***p*** ** < 0.001**	

Note

Bolded values indicate significant comparisons.

## APPENDIX 3

### Statistics comparing models for speed and pupation height with and without genotype as a nested term within population

#### 1

**Table:**

Trait	Model	*df*	AIC	BIC	χ^2^	*p*
Speed	Population	6	4168.9	4194.3		
	Population + population(genotype)	5	3914.2	3944.6	256.74	<0.001
Pupation height	Population	6	7193.9	7219.0		
	Population + population(genotype)	5	7050.9	7080.9	145.09	<0.001

## APPENDIX 4

### Statistical tests of individual comparisons of pupation height between populations, using Tukey's HSD

#### 1

**Table:**

	*D. mojavensis*				*D. arizonae*
	Baja	Catalina Island	Mojave	Sonora	Sonora
*D. mojavensis*					
Baja					
Catalina Island	***z*** ** = 5.336, ** ***p*** ** < 0.001**				
Mojave	*z* = 1.375, *p* = 0.628	***z*** ** = 9.163, ** ***p*** ** < 0.001**			
Sonora	*z* = 2.430, *p* = 0.099	***z*** ** = 10.838, ** ***p*** ** < 0.001**	*z* = 1.740, *p* = 0.392		
*D. arizonae*					
Sonora	*z* = 2.557, *p* = 0.072	***z*** ** = 11.163, ** ***p*** ** < 0.001**	*z* = 1.973, *p* = 0.264	*z* = 0.190, *p* = 0.999	

Note

Bolded values indicate significant comparisons.

## APPENDIX 5

### Statistical tests of individual comparisons of third‐instar speed between parental populations and F_1_ crosses, using Tukey's HSD

#### 1

**Table:**

	Catalina Island (CI)	CI × MJ122	MJ122 × CI	Sonora (MJ122)
Catalina Island (CI)				
CI x MJ122	***z*** ** = 4.672, ** ***p*** ** < 0.001**			
MJ122 × CI	*z* = 1.538, *p* = 0.399	*z* = 1.211, *p* = 0.606		
Sonora (MJ122)	***z*** ** = 6.648, ** ***p*** ** < 0.001**	*z* = 2.478, *p* = 0.059	*z* = 2.444, *p* = 0.064	

Note

Bolded values indicate significant comparisons.

## APPENDIX 6

### Statistical tests of individual comparisons of pupation height between parental populations and F_1_ crosses, using Tukey's HSD

#### 1

**Table:**

	Catalina Island (CI)	CI × MJ122	MJ122 × CI	Sonora (MJ122)
Catalina Island (CI)				
CI × MJ122	*z* = 1.348, *p* = 0.519			
MJ122 × CI	*z* = 1.811, *p* = 0.257	*z* = 0.934, *p* = 0.778		
Sonora (MJ122)	***z*** ** = 10.904, ** ***p*** ** < 0.001**	***z*** ** = 11.956, ** ***p*** ** < 0.001**	***z*** ** = 6.087, ** ***p*** ** < 0.001**	

Note

Bolded values indicate significant comparisons.
